# Neutrophil-to-Lymphocyte Ratio, Bone Marrow, and Visceral Fat Metabolism as Predictors of Future Cardiovascular Disease in an Asymptomatic Healthy Population

**DOI:** 10.3390/jcm14196709

**Published:** 2025-09-23

**Authors:** Soo Jin Lee, Jahae Kim, Ji Young Kim, Jin Chul Paeng, Yun Young Choi, Young Seo Kim, Kang-Ho Choi, Jeong-Min Kim, Nayeon Choi, Jiyeong Kim

**Affiliations:** 1 Department of Nuclear Medicine, Hanyang University Medical Center, Hanyang University College of Medicine, 222-1 Wangsimni-ro, Seongdong-gu, Seoul 04763, Republic of Korea; leesoojin@hanyang.ac.kr (S.J.L.); yychoi@hanyang.ac.kr (Y.Y.C.); 2 Department of Nuclear Medicine, Chonnam National University Hospital and Medical School, 160, Baekseo-ro, Dong-gu, Gwangju 61469, Republic of Korea; jhbt0607@hanmail.net; 3 Department of Nuclear Medicine, Seoul National University Hospital, Seoul National University College of Medicine, 101, Daehak-ro, Jongno-gu, Seoul 03080, Republic of Korea; paengjc@snu.ac.kr; 4 Department of Neurology, Hanyang University Medical Center, Hanyang University College of Medicine, 222-1 Wangsimni-ro, Seongdong-gu, Seoul 04763, Republic of Korea; kimys1@hanyang.ac.kr; 5 Department of Neurology, Chonnam National University Hospital and Medical School, 160, Baekseo-ro, Dong-gu, Gwangju 61469, Republic of Korea; ckhchoikang@hanmail.net; 6 Department of Neurology, Seoul National University Hospital, Seoul National University College of Medicine, 101, Daehak-ro, Jongno-gu, Seoul 03080, Republic of Korea; bellokim1@gmail.com; 7 Biostatics Lab, Medical Research Collaborating Center, Industry-University Cooperation Foundation, Hanyang University, 222-1 Wangsimni-ro, Seongdong-gu, Seoul 04763, Republic of Korea; nayeon@hanyang.ac.kr; 8 Department of Pre-Medicine, College of Medicine, Hanyang University, 222-1 Wangsimni-ro, Seongdong-gu, Seoul 04763, Republic of Korea; kimzi@hanyang.ac.kr

**Keywords:** neutrophils, lymphocytes, positron-emission-tomography, cardiovascular risk, bone marrow, visceral fat

## Abstract

**Background/Objectives:** The neutrophil-to-lymphocyte ratio (NLR), a marker of systemic inflammation, is a known predictor of cardiovascular disease and overall mortality. We examined the relationship between the NLR and the metabolic activity of hematopoietic organs and visceral fat, and their association with the risk of atherosclerotic cardiovascular disease (ASCVD) in an asymptomatic healthy population. **Methods:** We retrospectively analyzed individuals who underwent F-18-fluorodeoxyglucose (FDG) positron emission tomography/computed tomography (PET/CT) as part of their health check-ups. Metabolic activity was quantified using standardized uptake values (SUVs) from the lumbar vertebral bone marrow, spleen, visceral, and subcutaneous fat, normalized to target-to-background ratios (TBRs) using the superior vena cava. NLR was calculated from absolute neutrophil and lymphocyte counts. Correlations between NLR, clinical parameters, organ TBRs, and ASCAD risk were analyzed. **Results:** Among 303 participants from three hospitals, the median NLR was 1.5 (range: 0.5–5.55). NLR showed weak correlation with the TBRs of bone marrow, visceral fat, and subcutaneous fat, as well as high-density lipoprotein cholesterol and body mass index (BMI). In logistic regression analysis adjusted for age and sex, BMI and the TBRs of bone marrow and visceral fat were independent predictors of elevated NLR (≥ 1.5). When integrating these parameters, NLR demonstrated strong predictive performance for identifying a high ASCVD risk (≥20% over 10 years), with an area under the curve of 0.826. **Conclusions:** In an asymptomatic healthy population, NLR is associated with FDG metabolic parameters of hematopoietic organs and adipose tissue. These combined measures may serve as valuable marker for identifying individuals at elevated ASCVD risk.

## 1. Introduction

Atherosclerosis is a chronic inflammatory vascular disease characterized by the accumulation of plaque on the inner walls of blood vessels. It is the primary cause of atherosclerotic cardiovascular disease (ASCVD), leading to clinically significant events such as myocardial infarction and stroke.

Neutrophils play a crucial role in the pathogenesis of atherosclerosis, a process driven by systemic inflammation [1]. The neutrophil-to-lymphocyte ratio (NLR), a simple, readily accessible, and highly reproducible surrogate marker of inflammatory status, has proven to be a robust prognostic indicator of cancer, infection, and all-cause mortality [2,3,4]. It is a significant predictor of mortality and major adverse cardiovascular events in patients with acute coronary syndromes and heart failure [5,6]. According to a meta-analysis of eight studies of 9406 patients with acute coronary syndrome [5], a higher pretreatment NLR was associated with higher in-hospital mortality (odds ratio [OR] 6.39, 95% confidence interval [CI] 1.49–27.38). In patients with acute heart failure, an NLR cutoff value of 5.0 increased the risk of in-hospital and post-discharge three-year mortality [6].

Recent studies have shown that the prognostic value of NLR extends beyond its association with cardiovascular events and reflects the underlying chronic low-grade inflammatory conditions that influence atherosclerotic plaque instability and progression, such as diabetes [7,8], hypertension [9], obesity [10,11], and smoking [8,12]. In health cohort studies, a higher NLR was associated with the prevalence and incidence of type 2 diabetes [7] and hypertension [9]. Smoking cessation significantly lowered the median NLR from 1.8 (interquartile range [IQR] 1.56–2.5) to 1.7 (IQR 1.3–2.4) [13].

Consequently, NLR can be easily applied to clinical health assessments. The normal range for the NLR has not yet been established; however, it is generally considered to be between 0.78 and 3.53 [14]. Forget et al. [14] have reported that non-geriatric adults have an average NLR of 1.65.

Vascular inflammation and its association with the immune system are mediated by the activation of hematopoietic organs, with neutrophils derived from hematopoietic stem cells in the bone marrow. Bone marrow is activated in response to various normal and pathological stimuli, including inflammation, cancer, and infection. This is reflected in the glucose metabolism, which can be monitored using F-18-fluorodeoxyglucose positron emission tomography/computed tomography (^18^F-FDG PET/CT) [15]. Murata et al. [15] have found that FDG uptake by the bone marrow significantly correlates with various hematological parameters, including neutrophil count.

^18^F-FDG PET/CT is a non-invasive imaging modality that detects not only hematopoiesis but also inflammatory processes throughout the whole body [16]. It is widely used in clinical research to assess the inflammation of vascular structures such as the carotid arteries, aorta, and coronary arteries [17,18,19]. Additionally, this imaging modality quantifies the activation of hematopoietic organs, including bone marrow, spleen, and liver, in systemic conditions such as infections and cancer staging [20,21]. In individuals with atherosclerosis, increased metabolic activity in the spleen and bone marrow has been shown to predict subsequent cardiovascular events, indicating the crucial role of immune system activation in the progression of the disease [22,23,24]. Devesa et al. [25] have reported that bone marrow uptake on FDG PET/CT was associated with metabolic syndrome and with the prevalence of its components (obesity, hypertension, and glucose metabolism) in 745 healthy individuals, even in the absence of circulating systemic inflammation (below-median high-sensitivity C-reactive protein).

Although elevated NLR has been linked to cardiovascular events and prognosis [2], its relationship with the metabolic activity of hematopoietic organs in healthy individuals and its correlation with atherosclerotic CVD (ASCVD) risk remains unclear. In clinical practice, there is an increasing interest in assessing health status and predicting disease risk in individuals without specific medical conditions. With the aging population, there is growing interest in assessing the risk of ASCVD in healthy individuals to facilitate disease prevention.

This study aimed to evaluate the association between NLR and FDG metabolic parameters in a generally healthy population and explore their potential as indicators for monitoring increased ASCVD risk.

## 2. Materials and Methods

### 2.1. Study Participants

This study retrospectively reviewed ^18^F-FDG PET/CT assessments from routine health check-ups between December 2016 and September 2022 at three university hospitals in South Korea. A total of 303 participants (177 men and 126 women; mean age 57.4 ± 9.6 years) who were taking anti-diabetic, cholesterol-lowering, and antihypertensive medications were consecutively and equally enrolled in this study. Individuals who underwent a whole-body PET/CT scan during a health check-up at each hospital were included in this study. The exclusion criteria were as follows: (1) history of a malignancy under treatment, (2) acute cardiovascular or stroke history in less than 6 months, or (3) inadequate PET/CT image for analysis.

### 2.2. Clinical and Laboratory Parameters

We reviewed participants’ clinical data, including their vascular risk factor profiles and medical history. Three neurologists at each hospital reviewed their medical records at each hospital. Additionally, laboratory data, including white blood cell (WBC) count, neutrophils, lymphocytes, total cholesterol, triglycerides, low-density lipoprotein cholesterol, high-density lipoprotein (HDL) cholesterol, and fasting blood glucose levels, were collected. NLR was determined by dividing the absolute neutrophil count by the absolute lymphocyte count. The 10-year ASCVD risk was estimated using the ASCVD risk score calculator based on the 2019 American College of Cardiology/American Heart Association guidelines [26]. These guidelines provide an evidence-based framework for comprehensive risk factor management to reduce CVD incidence. The predicted 10-year ASCVD risk was categorized into low-risk (<5%), borderline risk (5–7.4%), intermediate risk (7.5–19.9%), and high risk (≥20%). Among these, the primary outcome of interest was patients in the high-risk group who were likely to develop CVD within the next 10 years.

### 2.3. ^18^F-FDG PET/CT Imaging and Metabolic Parameters

**Figure 1:**
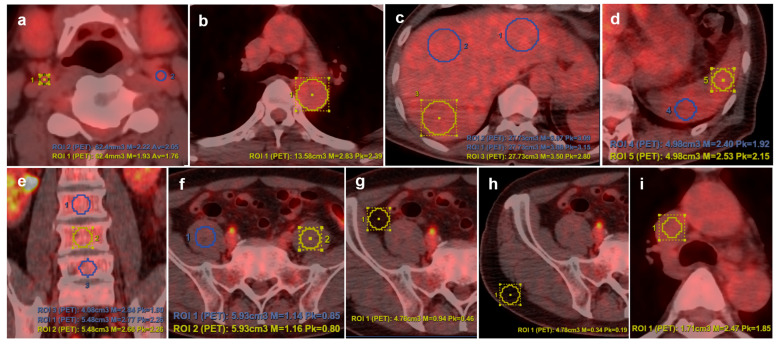
Representative volume of interest (VOI) placement for determining the metabolic parameters of various organs on ^18^F-FDG PET/CT. VOIs were drawn at following organs: (**a**) proximal internal carotid artery (pICA), (**b**) thoracic aorta, (**c**) liver, (**d**) spleen, (**e**) lumbar vertebrae (L3–L5), (**f**) psoas muscle, (**g**) visceral fat, (**h**) subcutaneous fat, and (**i**) superior vena cava (SVC) (for reference blood pool activity). SUV_max_ or SUV_peak_ values were averaged as indicated, and target-to-background ratios (TBRs) were calculated for analysis.

^18^F-FDG PET/CT scans were performed using different dedicated PET/CT scanners at each hospital (Biograph 6, Siemens Medical Systems, Knoxville, TN; Discovery 600 and Discovery STE System, GE Healthcare, Milwaukee, WI, USA). All participants fasted for more than 6 h before ^18^F-FDG administration. Approximately 60 min after intravenous injection of ^18^F-FDG, CT images were acquired, followed immediately by whole-body PET scans, covering the area from the base of the skull to the upper thigh. Image reconstruction was performed using a standard iterative algorithm (OSEM) and analyzed at a dedicated workstation equipped with fusion software for displaying CT, PET, and PET/CT images (MMWP, Siemens Medical Systems; Advantage 4.6 workstation, GE Medical System). At each hospital, a nuclear medicine physician with over 15 years of experience (Soo Jin Lee, Jahae Kim, and Ji Young Kim) reviewed PET-CT images. To ensure the consistency and comparability of data across hospitals, the three physicians underwent harmonization training before the start of the study. The training focused on standardizing the procedure for selecting and delineating the volume of interest on PET/CT images to minimize inter-observer variability in data acquisition. To evaluate glucose metabolism in the vessel walls and metabolically active organs, including the proximal internal carotid artery (pICA), thoracic aorta, spleen, liver, bone marrow (L3–5), psoas muscle, visceral fat, and abdominal subcutaneous fat, spherical volumes of interest were utilized to calculate the maximum and peak standardized uptake values (SUV_max_ and SUV_peak_). These were defined as follows: SUV = (regional activity [mCi/mL])/(injected dose [mCi]/body weight [g]). SUV_max_ represents the highest uptake value within a specific area, while SUV_peak_ is the average uptake value within a 1 cm^3^ sphere centered on the maximum value of the sphere [4,27]. SUV_max_ was measured for the pICA and thoracic aorta, whereas SUV_peak_ was measured for other regions. Each SUV_max_ and SUV_peak_ were normalized by dividing them by the background SUV obtained from the superior vena cava, resulting in the target-to-background ratio (TBR), which was used for analysis (Figure 1, Supplementary Table S1).

### 2.4. Statistical Analysis

Continuous data were presented as means ± standard deviation (SD) or medians (interquartile range) and were compared between groups using either Student’s *t*-tests or Wilcoxon rank-sum tests, as appropriate, following normality tests. Categorical data were expressed as frequencies (%) and were analyzed using the chi-square test. To identify independent predictors of higher NLR, Spearman’s rank correlation coefficients were used for correlation analysis, while simple and multiple logistic regression analyses were performed. The areas under the receiver operating characteristic curves (AUCs) were calculated to evaluate the predictive performance of three models for assessing high 10-year ASCVD risk. Model 1 included NLR alone. Model 2 added clinical and laboratory parameters, including sex, smoking status, body mass index (BMI), and HDL cholesterol, to Model 1. Model 3 further added SUV_peak_ of the bone marrow and visceral fat to Model 2. Statistical analyses were performed using SAS (version 9.4, SAS Institute Inc., Cary, NC, USA) and Jamovi (version 2.3.28). A two-tailed *p*-value of 0.05 was considered statistically significant.

## 3. Results

### 3.1. Baseline Characteristics of Study Participants

This analysis included a total of 303 participants (177 men and 126 women), with a mean age of 57.4 ± 9.6 years (range: 40.0–83.0 years). In our cohort, the mean NLR was 1.69 (SD 0.76, range 0.48–5.55) with a median of 1.5. Based on the median NLR value, participants were categorized into two groups: high NLR (≥ 1.5, n = 159) and low NLR (<1.5, n = 144). Table 1 and Supplementary Table S2 present the clinical and metabolic characteristics, along with the 10-year ASCVD risk score and risk category, stratified by NLR level. Compared to the low NLR (<1.5) group, participants in the high NLR (≥1.5) group were more likely to be male, smokers, and have a higher BMI, WBC count, neutrophil count, and a lower lymphocyte count and HDL cholesterol levels (all *p* < 0.05). Additionally, the high NLR group exhibited a higher SUV_peak_ for bone marrow metabolism at the L3–5 level (*p* = 0.025) and a higher 10-year ASCVD risk score (*p* = 0.014).

### 3.2. Correlation Between NLR and Various Clinical and Metabolic Parameters

We examined the associations between NLR and various clinical and metabolic parameters. BMI, WBC count, neutrophil count, and metabolic activity of bone marrow, visceral fat, and subcutaneous fat demonstrated significant positive correlations with NLR, whereas lymphocyte count and HDL cholesterol level showed negative correlations with NLR (all, *p* < 0.05) (Supplementary Table S3). After adjusting for age and sex, logistic regression analysis identified the following independent risk factors for predicting high NLR (≥ 1.5): smoking (OR = 1.085, *p* = 0.011), higher BMI (OR = 1.001, *p* < 0.001), increased bone marrow metabolism (L3–5) (OR = 2.350, *p* = 0.012), and visceral fat metabolism (OR = 12.230, *p* = 0.003). Although HDL cholesterol was initially identified as a significant risk factor, it lost statistical significance after adjusting for age and sex. Given the definition of NLR, it was expected that WBC, neutrophil, and lymphocyte counts would also independently predict a high NLR (Table 2).

### 3.3. Predictive Value of NLR and Various Clinical and Metabolic Parameters for 10-Year ASCVD Risk Score

**Figure 2:**
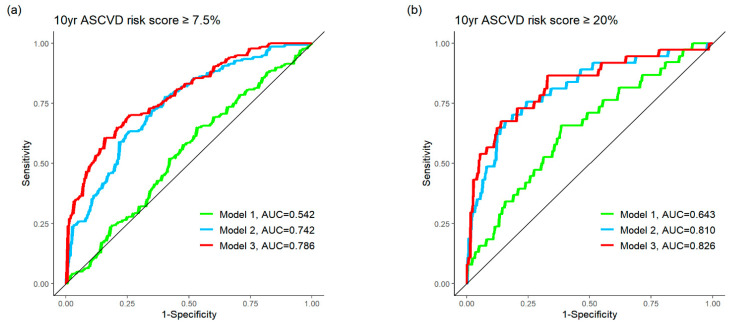
Comparison of ROC curve analyses of the different logistic regression models according to different 10-year ASCVD risk score ((**a**): ≥7.5%; (**b**): ≥20%). Model 1: NLR; Model 2: Model 1 + clinical parameter (sex, smoking, BMI, HDL); Model 3: Model 2 + PET metabolic parameter (SUV_peak_ of vertebra + SUV_peak_ of visceral fat). ROC, receiver–operating characteristic curve; ASCVD, atherosclerotic cardiovascular disease; NLR; neutrophil-to-lymphocyte ratio; BMI, body mass index; HDL, high-density lipoprotein cholesterol; AUC, area under curve.

Three logistic regression models were developed to assess the predictive value of NLR, along with clinical and metabolic parameters, in identifying 10-year ASCVD risk categories using AUC comparison. Among these models, Model 3, which included NLR, sex, smoking status, BMI, HDL, SUV_peak_ of the vertebrae, and visceral fat, demonstrated the highest AUC. This finding suggests that incorporating NLR with clinical and metabolic parameters derived from ^18^F-FDG PET/CT improves the model’s ability to accurately identify participants with 10-year ASCVD risk scores of ≥7.5% (intermediate-to-high-risk subgroup) and ≥20% (high-risk subgroup) (Table 3 and Figure 2). In addition to the well-established clinical characteristics of a high NLR, factors such as male sex, smoking, obesity, dyslipidemia, and increased metabolism of bone marrow and visceral fat on ^18^F-FDG PET/CT were associated with a higher likelihood of developing ASCVD in the future.

## 4. Discussion

This study examined the association between NLR and various metabolic parameters from ^18^F-FDG PET/CT with regard to ASCVD risk among asymptomatic healthy individuals. An elevated NLR (≥1.5) correlated with male sex, smoking status, BMI, and SUVs of bone marrow, visceral fat, and subcutaneous fat, as well as with the 10-year ASCVD risk score. Logistic regression analysis, adjusted for age and sex, identified smoking, BMI, and SUVs of bone marrow and visceral fat as significant predicting factors for higher NLR. When combined with these factors, NLR demonstrated strong predictive performance, with an AUC of 0.826 for identifying individuals at high risk (10-year ASCVD risk score ≥ 20%) and 0.786 for those at intermediate risk (risk score ≥ 7.5%).

Our findings indicate that elevated NLR is associated with well-established cardiovascular risk factors, including male sex, smoking, increased BMI, and lower HDL cholesterol. These findings are consistent with previous research in this field [13,28,29,30,31,32]. It has been suggested that bone marrow activation, triggered by various cardiovascular risk factors, leads to the release of inflammatory cells that contribute to atherosclerotic inflammation and CVD [25,32]. Based on this association, it can be hypothesized that elevated NLR, a marker of systemic inflammation, correlates with increased bone marrow metabolism, indicating bone marrow activation, as well as increased vessel wall metabolism, reflecting vascular inflammation. Our findings suggest a potential link between NLR and both bone marrow activation and adipose tissue inflammation, as indicated by elevated ^18^F-FDG uptake. However, no significant relationship was observed between NLR and vascular inflammation. Devesa et al. reported a correlation between bone marrow activation and early atherosclerosis, characterized by high arterial metabolic activity measured by SUV_max_ across six vascular regions [25]. In our study, the metabolic activity of the pICA and thoracic aorta was assessed using the TBR value of SUV_max_. Regarding high and low NLR subgroups, no significant differences were observed in the metabolic activity of these vascular regions (Table 1 and Supplementary Table S2). We believe these results may be due to the overall healthy status of the study population. In this study, the median NLR in the high NLR group was 2.1 (range: 1.3–5.5), while in the low NLR group, it was 1.2 (range: 0.5–1.5). The median NLR value in the high NLR group was lower than those reported in several studies involving patients with CVD or associated risk factors. For instance, Kim et al. reported a mean NLR value of 3.1 in patients with type 2 diabetes mellitus and significant CVD, defined as at least one vessel with >50% stenosis in major branches, as determined by coronary angiography [33]. Additionally, a retrospective analysis of patients with new-onset acute coronary syndrome showed that an NLR ≥ 6.94 was independently associated with coronary lesion severity [34]. In the present study, 18 participants had an NLR > 3.1, with mean metabolic activity values of 1.43 and 1.73 for pICA and thoracic aorta, respectively, which were not significantly different from those in the high NLR group (>1.5). Furthermore, several studies have suggested that splenic metabolic activity on ^18^F-FDG PET/CT may predict the risk of future cardiovascular events and systemic inflammation [22]. Similarly to our findings on vascular metabolism, splenic metabolism exhibited a comparable trend.

Various studies have demonstrated a correlation between NLR and cardiovascular risk in patients with hypertension, diabetes mellitus, or obesity [4,35,36]. Dong et al. suggested that elevated NLR was independently associated with increased all-cause and cardiovascular mortality in patients with diabetes [35]. They reported that hyperglycemia affects both the quantity and function of circulating neutrophils. This phenomenon was observed specifically in patients with type 2 diabetes mellitus [8], where the expression of activation markers on neutrophil membranes differs from that in healthy controls, ultimately leading to systemic inflammation and endothelial damage through oxidative stress [12]. Similarly, Bagyura et al. identified an independent association between subclinical chronic systemic inflammation and subclinical coronary disease in individuals with obesity [36]. To assess the impact of diabetes mellitus on our study cohort, we performed a subgroup analysis. Compared with our overall cohort, individuals with diabetes (Supplementary Table S4A,B) with a high NLR exhibited a significantly increased 10-year ASCVD risk (median 17.5 vs. 8.5, *p* = 0.037). Non-diabetic individuals (Supplementary Table S5A,B) with a high NLR demonstrated trends similar to those observed in the overall cohort. In addition to the previously identified PET–metabolic parameters, metabolic activity in the spleen and psoas muscle emerged as a significant predictor of an elevated NLR. These findings suggest that PET-derived metabolic parameters may play a particularly prominent role in identifying elevated NLR in non-diabetic individuals, highlighting the potential utility of PET imaging for early risk stratification in asymptomatic, healthy populations. In our study, the overall prevalence of hypertension, diabetes mellitus, and hyperlipidemia among all participants was 31.4%, 16.2%, and 36.9%, respectively. Furthermore, no significant differences were observed between the low NLR and high NLR subgroups in the prevalence of hypertension (30.8% vs. 31.9%), diabetes (14.5% vs. 18.1%), and hyperlipidemia (38.4% vs. 35.4%). These findings may be due to the relatively asymptomatic nature of the study population.

Variations in mean NLR have been observed across different racial populations without underlying disease (Supplementary Table S6 [3,11,14,37,38,39,40,41,42,43,44]). In studies from the United States [37,38] that included ethnically diverse populations, the non-Hispanic Black group had a lower NRL than the non-Hispanic White, Hispanic, and other groups (1.78 for non-Hispanic Black, 2.27 for non-Hispanic White, 2.10 for Hispanic, and 2.03 for other groups). This may be due to genetic factors, lifestyle, or differences in the prevalence of chronic diseases. The NRL tended to increase with age. In a Sicilian supercentenarian study [39], the older age group of 65–90 years had a higher mean NLR than the adult group of below 65 years (1.98 ± 0.84 vs. 1.65 ± 0.64). Additionally, the average NLR in males is slightly higher than in females. The cause of the difference in NLR between males and females is not yet clear, but differences in baseline inflammation between the sexes may be a contributing factor [45].

The mean NLR for our study participants, who were predominantly Koreans, was 1.5. This was lower than the reported mean of 2.0 for individuals without significant coronary artery disease [33] and the mean value of 1.92 in the lower tertile for coronary artery events [46]. It was also lower than the normal range observed in other populations, such as 1.76 for non-Hispanic Black individuals and 2.08 for Hispanic participants [37]. These differences suggest that NLR study results may vary depending on the racial composition of the study population.

This study has several limitations due to its retrospective design. First, detailed data on medication history, including specific classes or active ingredients of cholesterol-lowering, anti-diabetic, or antihypertensive agents, were not collected, which may have influenced the observed associations. As this was a retrospective study, detailed information on medication use could not be collected during the data review. The study population consisted of an asymptomatic healthy cohort. This means that even in the presence of erlying chronic diseases such as diabetes or hypertension, these were well controlled and did not represent severe or long-standing pathology. Therefore, we believe that the potential for medication use as a significant confounding factor was minimal.

Second, inflammatory markers such as C-reactive protein were not assessed, limiting our ability to evaluate the independent contribution of systemic inflammation to metabolic parameters. Third, the multicenter nature of the study required the use of target-to-background ratio (TBR) for SUV evaluation of metabolic activity on ^18^F-FDG PET/CT, resulting in relatively low absolute values and modest differences between high and low metabolic activity groups. Fourth, as this was an observational study, a causal relationship between NLR-driven inflammation and vascular or metabolic dysfunction could not be established. Experimental studies, such as neutrophil depletion models, are required to explore these mechanistic pathways.

## 5. Conclusions

The NLR is indeed an important marker associated with ASCVD risk, even in asymptomatic healthy populations. We developed a comprehensive model incorporating NLR and various clinical and metabolic parameters, demonstrating its potential for improved ASCVD risk stratification. By integrating ^18^F-FDG PET/CT-derived metabolic data with traditional risk factors, this approach enables more precise identification of individuals at higher risk. Bone marrow and visceral fat metabolism on ^18^F-FDG PET/CT may serve as semi-quantitative predictors of future ASCVD risk in asymptomatic relatively healthy population. Despite certain limitations, our findings provide valuable insights into the relationship between NLR and FDG PET-derived metabolic parameters. This study combined simple blood tests and advanced imaging data reflecting systemic inflammation to assess cardiovascular risk, which could be useful for personalized preventive healthcare.

Future prospective studies, including diverse ethnic populations and comprehensive assessment of inflammatory markers and medication effects, are warranted to validate these observations and to explore their potential in guiding personalized preventive strategies in clinical practice.

## Figures and Tables

**Table 1:** Clinical characteristics of participants based on NLR (median value).

	Low (<1.5, n = 159)	High (≥1.5, n = 144)	*p*-Value
**Clinical parameters**			
Age, years	57.5 ± 9.0	57.4 ± 10.2	0.936
Sex, male	78 (49.1)	99 (68.8)	<0.001
Hypertension	49 (30.8)	46 (31.9)	0.833
Diabetes mellitus	23 (14.5)	26 (18.1)	0.397
Hyperlipidemia	61 (38.4)	51 (35.4)	0.596
Smoking	25 (15.8)	42 (29.2)	0.005
Body mass index	24.0 (22.0–26.2)	25.3 (23.2–27.9)	0.001
White blood cell, ×10^9^/L	4800.0 (4100.0–5900.0)	5950.0 (5050.0–6900.0)	<0.001
Neutrophil, ×10^9^/L	2309.1 (1876.5–2867.0)	3630.6 (3099.6–4460.7)	<0.001
Lymphocyte, ×10^9^/L	2085.4 ± 577.9	1741.0 ± 491.0	<0.001
Total cholesterol, mg/dL	201.9 ± 39.7	193.0 ± 43.5	0.066
Triglyceride, mg/dL	107.0 (81.0–149.0)	120.0 (89.0–171.0)	0.081
LDL cholesterol, mg/dL	123.2 ± 32.0	119.4 ± 34.0	0.310
HDL cholesterol, mg/dL	54.0 (46.0–64.0)	49.0 (42.0–59.0)	0.003
Fasting blood glucose, mg/dL	98.0 (91.0–108.0)	101.0 (90.0–110.0)	0.424
**Metabolic parameters [SUV, TBR]**			
pICA	1.43 (1.28–1.65)	1.43 (1.31–1.67)	0.937
Thoracic aorta	1.84 (1.66–2.02)	1.87 (1.68–2.06)	0.669
Spleen	1.28 (1.18–1.44)	1.35 (1.17–1.53)	0.125
Liver	1.97 (1.79–2.17)	1.97 (1.80–2.21)	0.542
L3–5	1.23 (1.05–1.49)	1.33 (1.13–1.64)	0.025
Psoas	0.58 (0.47–0.69)	0.59 (0.51–0.68)	0.266
Visceral fat	0.32 (0.25–0.42)	0.35 (0.26–0.47)	0.053
SubQ fat	0.18 (0.14–0.22)	0.19 (0.14–0.25)	0.130
**10-year ASCVD risk ^†^**	5.7 (2.3–10.9)	8.1 (3.0–16.3)	0.014
**10-year ASCVD risk category**			0.098
Low	70 (44.0)	53 (37.0)	
Borderline	22 (13.8)	17 (11.9)	
Intermediate	54 (34.0)	48 (33.6)	
High	13 (8.2)	25 (17.5)	

NLR, neutrophil-to-lymphocyte ratio; LDL, low-density lipoprotein; HDL, high-density lipoprotein; TBR, targe-to-background ratio; SubQ, subcutaneous; pICA, proximal intracranial artery; ASCVD, atherosclerotic cardiovascular disease. Data are presented as means ± standard deviations, median (Q1–Q3), or frequencies (%) unless otherwise indicated. *p*-values are based on Pearson’s chi-square test, Student’s *t*-test, or Wilcoxon rank-sum test. ^†^ 10-year risk for ASCVD is categorized as low-risk (<5%), borderline risk (5% to 7.4%), intermediate risk (7.5% to 19.9%), high risk (≥20%) by American College of Cardiology ASCVD risk estimator.

**Table 2:** Logistic regression model predicting high NLR (≥1.5) in the population of health examination.

	No Adjustment		Adjustment for Age and Sex	
	OR (95% CI)	*p*-Value	OR (95% CI)	*p*-Value
**Clinical parameter**				
Age	0.999 (0.976–1.023)	0.936		
Sex, male	2.285 (1.428–3.655)	<0.001		
Hypertension	1.054 (0.648–1.713)	0.833	0.950 (0.567–1.594)	0.847
Diabetes mellitus	1.303 (0.706–2.405)	0.397	1.249 (0.667–2.337)	0.487
Hyperlipidemia	0.881 (0.552–1.407)	0.596	0.908 (0.563–1.466)	0.694
Smoking	2.191 (1.253–3.828)	0.006	1.663 (0.893–3.095)	0.109
Body mass index	1.103 (1.037–1.173)	0.002	1.085 (1.019–1.155)	0.011
White blood cell	1.001 (1.000–1.001)	<0.001	1.001 (1.000–1.001)	<0.001
Neutrophil	1.002 (1.002–1.003)	<0.001	1.002 (1.002–1.003)	<0.001
Lymphocyte	0.999 (0.998–0.999)	<0.001	0.999 (0.998–0.999)	<0.001
Total cholesterol	0.995 (0.989–1.000)	0.067	0.996 (0.990–1.002)	0.188
Triglyceride	1.002 (0.999–1.006)	0.146	1.001 (0.998–1.004)	0.522
LDL cholesterol	0.996 (0.990–1.003)	0.309	0.997 (0.990–1.004)	0.443
HDL cholesterol	0.972 (0.955–0.990)	0.003	0.982 (0.963–1.002)	0.081
Fasting blood glucose	1.000 (0.993–1.008)	0.905	1.000 (0.992–1.008)	0.931
**PET metabolic parameters**				
pICA	0.861 (0.421–1.761)	0.681	0.821 (0.393–1.713)	0.599
Thoracic aorta	0.876 (0.469–1.637)	0.679	0.922 (0.486–1.749)	0.805
Spleen	1.705 (0.676–4.302)	0.258	2.210 (0.849–5.752)	0.104
Liver	1.291 (0.611–2.727)	0.504	1.307 (0.603–2.831)	0.497
L3–5	1.921 (1.038–3.558)	0.038	2.350 (1.208–4.571)	0.012
Psoas	2.715 (0.636–11.581)	0.177	4.467 (0.962–20.732)	0.056
Visceral fat	7.570 (1.567–36.585)	0.012	12.230 (2.322–64.403)	0.003
SubQ fat	16.103(0.934–277.680)	0.056	10.896 (0.598–198.551)	0.107

NLR, neutrophil-to-lymphocyte ratio; LDL, low-density lipoprotein; HDL, high-density lipoprotein; PET, positron emission tomography; pICA, proximal intracranial artery; SubQ, subcutaneous.

**Table 3:** Comparison of predictive performance of logistic regression models for identifying 10-year ASCVD risk category ^†^.

	Intermediate and High Risk (≥7.5%)		High Risk (≥20%)	
Model	AUC (95% CI)	*p*-Value (Versus Ref.)	AUC (95% CI)	*p*-Value (Versus Ref.)
1. NLR (ref.)	0.542	NA	0.643	NA
2. NLR, sex, smoking, BMI, HDL	0.742	<0.001	0.810	0.002
3. NLR, sex, smoking, BMI, HDL, SUV_peak_ of vertebra, SUV_peak_ of visceral fat	0.786	<0.001	0.826	0.001

ASCVD, atherosclerotic cardiovascular disease; NLR, neutrophil-to-lymphocyte ratio; BMI, body mass index; HDL, high-density lipoprotein. ^†^ Ten-year risk for ASCVD is categorized as low-risk (<5%), borderline risk (5% to 7.4%), intermediate risk (7.5% to 19.9%), and high risk (≥20%) by American College of Cardiology ASCVD risk estimator.

## Data Availability

The datasets used and/or analyzed during the current study are available from the corresponding author upon reasonable request.

## Supplementary Materials

The following supporting information can be downloaded at https://www.mdpi.com/article/10.3390/jcm14196709/s1. Table S1: VOI placement and SUV measurement methods on ^18^F-FDG PET/CT. Table S2: Clinical characteristics of participants based on NLR (NLR < 3.0 vs. ≥ 3.0). Table S3: Spearman’s correlation between clinical parameters, PET metabolic parameters, and NLR. Table S4: (A) Clinical characteristics of diabetic participants based on NLR. (B) Logistic regression model predicting high NLR (≥1.5) in diabetic participants. Table S5: (A) Clinical characteristics of non-diabetic participants based on NLR. (B) Logistic regression model predicting high NLR (≥ 1.5) in non-diabetic participants. Table S6: Neutrophil-to-lymphocyte ratios of healthy individuals in the literature.
