# Supplementary material for: Neutrophil-to-Lymphocyte Ratio, Bone Marrow, and Visceral Fat Metabolism as Predictors of Future Cardiovascular Disease in an Asymptomatic Healthy Population
Source: J Clin Med. 2025 Sep 23;14(19):6709. doi: 10.3390/jcm14196709 (PMC12525092; doi:10.3390/jcm14196709)
Supplement: Supplementary file 1 [file jcm-14-06709-s001.zip › jcm-3853222-supplementary.pdf]

Supplement Table S1. VOI placement and SUV measurement methods on ^{18}F-FDG PET/CT.

Organ/ Region	VOI number	VOI diameter (cm)	Volume (cm^3)	Location	SUV metric
Proximal ICA (pICA)	2	1.0–1.5	0.5–2.0	Right & left pICA above carotid bifurcation	mean SUV_{max}
Thoracic aorta	1	Flexible	–	Between aortic arch and intrathoracic descending aorta	SUV_{max}
Liver	3	3.6–4.4	25–45	Right, middle, and left lobe (avoiding central vessels)	mean SUV_{peak}
Spleen	2	2.0–2.5	4–8	Two portions of spleen	mean SUV_{peak}
Bone marrow (L3–L5)	3	2.0–2.5	4–8	Central vertebral bodies	mean SUV_{peak}
Psoas muscle	2	2.0–2.5	4–8	Right & left psoas within 5 cm of iliac crest	mean SUV_{peak}
Visceral fat	1	2.0–2.5	4–8	Peritoneal/retroperitoneal area along psoas (avoiding bowel/vessels)	SUV_{peak}
Subcutaneous fat	1	2.0–2.5	4–8	Gluteal area	SUV_{peak}
Blood pool (SVC)	1	1.2–1.6	1–2	At the level of carina	SUV_{peak}

VOI; volume of interest, SUV; standardized uptake value, PET; positron emission tomography, pICA; proximal intracranial artery, SVC; superior vena cava.

Supplement Table S2. Clinical characteristics of participants based on NLR (NLR < 3.0 vs. ≥ 3.0).

	Low (< 3, n = 283)	High (≥ 3, n = 20)	*p*-Value
**Clinical parameters**			
Age, years	57.0 (50.0-64.0)	63.0 (50.0-70.5)	0.112
Sex, male	165 (58.3)	12 (60.0)	0.882
Hypertension	89 (31.5)	6 (30.0)	0.893
Diabetes mellitus	45 (15.9)	4 (20.0)	0.544
Hyperlipidemia	106 (37.5)	6 (30.0)	0.504
Smoking (n=302)	64 (22.7)	3 (15.0)	0.581
Body mass index	24.5 (22.3-27.0)	24.6 (23.9-27.2)	0.319
White blood cell, x10^9/L	5300.0 (4400.0-6500.0)	6450.0 (5600.0-8050.0)	< 0.001
Neutrophil, x10^9/L	2914.2±1000.6	5043.4±1399.9	< 0.001
Lymphocyte, x10^9/L	1903.8 (1542.8-2296.0)	1303.8 (1157.4-1584.2)	< 0.001
Total cholesterol, mg/dL	198.0±41.8	193.0±40.7	0.601
Triglyceride, mg/dL	113.5 (84.0-160.0)	110.5 (85.5-150.0)	0.884
LDL cholesterol, mg/dL	121.7±33.3	117.2±28.6	0.551
HDL cholesterol, mg/dL	52.0 (44.0-61.0)	54.0 (44.0-60.5)	0.839
Fasting blood glucose, mg/dL	99.0 (91.0-109.0)	101.5 (89.0-113.5)	0.941
**Metabolic parameters [SUV, TBR]**			
pICA	1.43 (1.29-1.65)	1.45 (1.27-1.66)	0.898
Thoracic aorta	1.85 (1.67-2.06)	1.73 (1.61-1.98)	0.159
Spleen	1.31 (1.18-1.49)	1.31 (1.16-1.41)	0.674
Liver	1.97 (1.79-2.18)	1.92 (1.81-2.20)	0.789
L3-5	1.28 (1.10-1.57)	1.26 (1.11-1.44)	0.830
Psoas	0.58 (0.49-0.69)	0.61 (0.49-0.69)	0.851
Visceral fat	0.33 (0.25-0.44)	0.32 (0.24-0.44)	0.953
SubQ fat	0.19 (0.14-0.24)	0.16 (0.13-0.25)	0.473
**10-year ASCVD risk**[†]	6.8 (2.4-13.1)	6.4 (3.3-25.6)	0.344
**10-year ASCVD risk category**			0.017
Low	115 (40.8)	8 (40.0)	
Borderline	36 (12.8)	4 (20.0)	
Intermediate	99 (35.1)	2 (10.0)	
High	32 (11.4)	6 (30.0)	

NLR; neutrophil-to-lymphocyte ratio, LDL; low-density lipoprotein, HDL; high-density lipoprotein, TBR; targe-to-background ratio, SubQ; subcutaneous, pICA; proximal intracranial artery, ASCVD; atherosclerotic cardiovascular disease. Data are presented as means ± standard deviations, median (Q1-Q3), or frequencies (%) unless otherwise indicated. *p*-Values are based on the Pearson's Chi-square test, Student's t-test, or Wilcoxon rank-sum test. [†] 10-year risk for ASCVD is categorized as: Low-risk (< 5%), Borderline risk (5% to 7.4%), Intermediate risk (7.5% to 19.9%), High risk (≥ 20%) by American College of Cardiology ASCVD risk estimator.

Supplement Table S3. Spearman's correlation between clinical parameters, PET metabolic parameters and NLR.

NLR					
**Clinical parameter**			**PET-Metabolic parameter**		
	ρ	*p* - value		ρ	*p* - value
**Age (yr)**	0.013	0.824	pICA	0.009	0.877
**BMI (kg/m2)**	0.120	0.036	Thoracic aorta	-0.023	0.687
**White blood cell**	0.356	< 0.001	Spleen	0.059	0.306
**Neutrophil**	0.713	< 0.001	Liver	0.013	0.815
**Lymphocyte**	- 0.484	< 0.001	L3-5	0.134	0.020
**Total cholesterol**	-0.112	0.052	Psoas	0.043	0.461
**Triglyceride**	0.037	0.521	Visceral fat	0.129	0.025
**HDL**	-0.135	0.019	SubQ fat	0.127	0.028
**LDL**	-0.073	0.206			
**Fasting blood glucose**	0.052	0.370			

PET; positron emission tomography, NLR; neutrophil-to-lymphocyte ratio, BMI; body mass index, HDL; high-density lipoprotein, LDL; low-density lipoprotein, pICA; proximal intracranial artery, SubQ; subcutaneous.

Supplement Table S4A. Clinical characteristics of diabetic participants based on NLR.

	Low (< 1.5, n = 23)	High (≥ 1.5, n = 26)	*p*-Value
**Clinical parameters**			
Age, years	57.1±8.1	60.3±9.9	0.218
Sex, male	14 (60.9)	17 (65.4)	0.744
Hypertension	11 (47.8)	17 (65.4)	0.215
Hyperlipidemia	16 (69.6)	12 (46.2)	0.098
Smoking	2 (8.7)	8 (30.8)	0.080
Body mass index	26.0±3.1	26.7±3.7	0.447
White blood cell, x10^9/L	5252.2±1512.6	6626.9±1592.1	0.003
Neutrophil, x10^9/L	2529.3±780.6	4096.8±1186.6	< 0.001
Lymphocyte, x10^9/L	2149.8±670.5	1870.7±478.1	0.097
Total cholesterol, mg/dL	168.0 (144.0-229.0)	165.0 (132.0-184.0)	0.429
Triglyceride, mg/dL	127.0 (96.0-204.0)	112.5 (83.0-179.0)	0.596
LDL cholesterol, mg/dL	100.0 (75.0-141.0)	95.5 (77.0-111.0)	0.595
HDL cholesterol, mg/dL	50.1±11.9	49.6±12.9	0.886
Fasting blood glucose, mg/dL	133.0 (116.0-177.0)	135.5 (117.0-155.0)	0.667
**Metabolic parameters [SUV, TBR]**			
pICA	1.37±0.21	1.31±0.22	0.400
Thoracic aorta	1.77 (1.67-1.93)	1.69 (1.57-1.91)	0.417
Spleen	1.26±0.24	1.20±0.19	0.406
Liver	1.98±0.37	1.89±0.22	0.348
L3-5	1.27±0.34	1.18±0.28	0.315
Psoas	0.57±0.15	0.55±0.11	0.516
Visceral fat	0.33±0.10	0.32±0.11	0.743
SubQ fat	0.19±0.07	0.18±0.08	0.657
**10-year ASCVD risk†**	8.5 (5.3-15.3)	17.5 (8.4-28.2)	0.037
**10-year ASCVD risk category**			0.444
Low	4 (17.4)	3 (11.5)	
Borderline	4 (17.4)	3 (11.5)	
Intermediate	11 (47.8)	10 (38.5)	
High	4 (17.4)	10 (38.5)	

NLR; neutrophil-to-lymphocyte ratio, LDL; low-density lipoprotein, HDL; high-density lipoprotein, TBR; targe-to-background ratio, SubQ; subcutaneous, pICA; proximal intracranial artery, ASCVD; atherosclerotic cardiovascular disease. Data are presented as means ± standard deviations, median (Q1-Q3), or frequencies (%) unless otherwise indicated. *p*-Values are based on the Pearson's Chi-square test, Student's t-test, or Wilcoxon rank-sum test. † 10-year risk for ASCVD is categorized as: Low-risk (< 5%), Borderline risk (5% to 7.4%), Intermediate risk (7.5% to 19.9%), High risk (≥ 20%) by American College of Cardiology ASCVD risk estimator.

Supplement Table S4B. Logistic regression model predicting high NLR (≥ 1.5) in diabetic participants.

	No Adjustment		Adjustment for age and sex	
	OR (95% CI)	*p*-Value	OR (95% CI)	*p*-Value
**Clinical parameter**				
Age	1.041 (0.977-1.110)	0.215		
Sex, male	1.214 (0.379-3.888)	0.744		
Hypertension	2.061 (0.653-6.507)	0.218	1.974 (0.599-6.506)	0.264
Hyperlipidemia	0.375 (0.116-1.216)	0.102	0.449 (0.128-1.572)	0.211
Smoking	4.667 (0.876-24.851)	0.071	7.692 (1.184-49.983)	0.033
Body mass index	1.069 (0.903-1.266)	0.439	1.102 (0.921-1.319)	0.287
White blood cell	1.001 (1.000-1.001)	0.008	1.001 (1.000-1.001)	0.005
Neutrophil	1.002 (1.001-1.003)	< 0.001	1.002 (1.001-1.003)	< 0.001
Lymphocyte	0.999 (0.998-1.000)	0.102	0.999 (0.998-1.000)	0.137
Total cholesterol	0.995 (0.983-1.007)	0.390	0.997 (0.985-1.010)	0.671
Triglyceride	0.998 (0.991-1.005)	0.616	1.000 (0.992-1.008)	0.920
LDL cholesterol	0.995 (0.980-1.011)	0.546	0.998 (0.982-1.015)	0.833
HDL cholesterol	0.997 (0.952-1.044)	0.883	1.002 (0.951-1.055)	0.949
Fasting blood glucose	0.995 (0.984-1.007)	0.422	0.998 (0.985-1.011)	0.784
**PET metabolic parameters**				
pICA	0.314 (0.022-4.458)	0.392	0.395 (0.023-6.872)	0.524
Thoracic aorta	0.300 (0.030-3.019)	0.307	0.411 (0.037-4.536)	0.468
Spleen	0.311 (0.021-4.720)	0.400	0.364 (0.023-5.828)	0.475
Liver	0.378 (0.053-2.673)	0.329	0.489 (0.063-3.770)	0.492
L3-5	0.379 (0.058-2.470)	0.311	0.499 (0.070-3.540)	0.487
Psoas	0.215 (0.002-20.448)	0.508	0.246 (0.002-25.686)	0.555
Visceral fat	0.405 (0.002-78.294)	0.737	0.233 (0.001-52.985)	0.598
SubQ fat	0.166 (< 0.001-384.078)	0.650	0.134 (< 0.001-483.745)	0.630

NLR; neutrophil-to-lymphocyte ratio, LDL; low-density lipoprotein, HDL; high-density lipoprotein, PET; positron emission tomography, pICA; proximal intracranial artery, SubQ; subcutaneous.

Supplement Table S5A. Clinical characteristics of non-diabetic participants based on NLR.

	Low (< 1.5, n = 136)	High (≥ 1.5, n = 118)	*p*-Value
**Clinical parameters**			
Age, years	57.6±9.2	56.7±10.2	0.510
Sex, male	64 (47.1)	82 (69.5)	< 0.001
Hypertension	38 (27.9)	29 (24.6)	0.544
Hyperlipidemia	45 (33.1)	39 (33.1)	0.995
Smoking (n=253)	23 (17.0)	34 (28.8)	0.025
Body mass index	23.6 (21.9-25.8)	24.7 (23.0-27.8)	0.002
White blood cell, x10^9/L	4800.0 (4100.0-5600.0)	5850.0 (5000.0-6700.0)	< 0.001
Neutrophil, x10^9/L	2266.4 (1855.8-2831.1)	3580.1 (3020.0-4247.8)	< 0.001
Lymphocyte, x10^9/L	2074.5±562.8	1712.4±491.2	< 0.001
Total cholesterol, mg/dL	205.5±36.3	198.5±41.3	0.151
Triglyceride, mg/dL	105.0 (77.5-140.0)	120.0 (90.0-163.0)	0.038
LDL cholesterol, mg/dL	126.0±30.1	123.5±32.6	0.530
HDL cholesterol, mg/dL	55.0 (46.5-64.0)	50.0 (43.0-59.0)	0.002
Fasting blood glucose, mg/dL	96.0 (90.0-104.0)	98.0 (89.0-106.0)	0.463
**Metabolic parameters [SUV, TBR]**			
pICA	1.44 (1.30-1.69)	1.46 (1.34-1.70)	0.595
Thoracic aorta	1.85 (1.66-2.08)	1.92 (1.71-2.10)	0.325
Spleen	1.29 (1.19-1.45)	1.37 (1.24-1.54)	0.018
Liver	1.98 (1.79-2.17)	2.03 (1.82-2.22)	0.207
L3-5	1.23 (1.07-1.49)	1.38 (1.17-1.70)	0.002
Psoas	0.58 (0.49-0.69)	0.60 (0.52-0.71)	0.102
Visceral fat	0.32 (0.25-0.42)	0.36 (0.26-0.48)	0.021
SubQ fat	0.18 (0.14-0.22)	0.20 (0.15-0.25)	0.042
**10-year ASCVD risk**[ǂ]	5.3 (2.1-10.4)	6.5 (2.7-13.4)	0.084
**10-year ASCVD risk category**			0.389
Low	66 (48.5)	50 (42.7)	
Borderline	18 (13.2)	15 (12.8)	
Intermediate	43 (31.6)	37 (31.6)	
High	9 (6.6)	15 (12.8)	

NLR; neutrophil-to-lymphocyte ratio, LDL; low-density lipoprotein, HDL; high-density lipoprotein, TBR; targe-to-background ratio, SubQ; subcutaneous, pICA; proximal intracranial artery, ASCVD; atherosclerotic cardiovascular disease. Data are presented as means ± standard deviations, median (Q1-Q3), or frequencies (%) unless otherwise indicated. *p*-Values are based on the Pearson's Chi-square test, Student's t-test, or Wilcoxon rank-sum test. ǂ 10-year risk for ASCVD is categorized as: Low-risk (< 5%), Borderline risk (5% to 7.4%), Intermediate risk (7.5% to 19.9%), High risk (≥ 20%) by American College of Cardiology ASCVD risk estimator.

Supplement Table S5B. Logistic regression model predicting high NLR (≥ 1.5) in non-diabetic participants.

	No Adjustment		Adjustment for age and sex	
	OR (95% CI)	*p*-Value	OR (95% CI)	*p*-Value
**Clinical parameter**				
Age	0.991 (0.966-1.017)	0.508		
Sex, male	2.562 (1.528-4.295)	< 0.001		
Hypertension	0.840 (0.479-1.474)	0.544	0.783 (0.427-1.437)	0.430
Hyperlipidemia	0.998 (0.591-1.686)	0.995	1.073 (0.622-1.852)	0.800
Smoking	1.971 (1.082-3.592)	0.027	1.308 (0.668-2.560)	0.434
Body mass index	1.106 (1.034-1.182)	0.004	1.082 (1.010-1.159)	0.025
White blood cell	1.001 (1.000-1.001)	< 0.001	1.001 (1.000-1.001)	< 0.001
Neutrophil	1.002 (1.002-1.003)	< 0.001	1.002 (1.002-1.003)	< 0.001
Lymphocyte	0.999 (0.998-0.999)	< 0.001	0.998 (0.998-0.999)	< 0.001
Total cholesterol	0.995 (0.989-1.002)	0.152	0.996 (0.990-1.003)	0.305
Triglyceride	1.003 (1.000-1.007)	0.077	1.002 (0.998-1.006)	0.376
LDL cholesterol	0.997 (0.990-1.005)	0.528	0.998 (0.989-1.006)	0.605
HDL cholesterol	0.968 (0.949-0.988)	0.002	0.980 (0.959-1.002)	0.072
Fasting blood glucose	1.004 (0.984-1.024)	0.704	0.998 (0.978-1.019)	0.881
**PET metabolic parameters**				
pICA	1.004 (0.468-2.153)	0.992	0.968 (0.442-2.122)	0.936
Thoracic aorta	1.014 (0.524-1.962)	0.968	1.101 (0.558-2.170)	0.781
Spleen	2.473 (0.881-6.941)	0.086	3.578 (1.219-10.498)	0.020
Liver	1.693 (0.741-3.870)	0.212	1.714 (0.731-4.021)	0.216
L3-5	2.535 (1.289-4.986)	0.007	3.129 (1.501-6.522)	0.002
Psoas	4.070 (0.853-19.428)	0.078	8.698 (1.573-48.082)	0.013
Visceral fat	11.124 (2.064-59.944)	0.005	21.787 (3.530-134.468)	< 0.001
SubQ fat	37.699 (1.507-942.988)	0.027	27.565 (1.004-757.094)	0.050

NLR; neutrophil-to-lymphocyte ratio, LDL; low-density lipoprotein, HDL; high-density lipoprotein, PET; positron emission tomography, pICA; proximal intracranial artery, SubQ; subcutaneous.

Supplement Table S6. Neutrophil-to-lymphocyte ratios of healthy individuals in literature.

Authors, years	Countries	Patients, mean age	n	NLR, Mean ± standard deviation
Imtiaz et al. 2012 [40]	Pakistan	Healthy individuals, 16 ~ 75 years	1,070	0.17-1.51 (n=357)<br>1.52-2.56 (n=361)<br>2.56-22.50 (n=352)
Azab et al. 2014 [37]	United States	NHANES survey (2007-2010)<br>average 47 years	9,427	2.15 for all<br>2.08 for Hispanic<br>2.24 for Non-Hispanic white<br>1.76 for Non-Hispanic Black<br>2.10 Other non-Hispanic
Lee et al. 2017 [41]	Korean	Health checkup<br>median 47 years	12,160	1.65 (0.107-3.193)
Nam et al. 2017 [42]	Korean	For health check up<br>49 years for CACS = 0<br>56 years for CACS > 0	599	1.59 ± 0.63 for CACS = 0<br>1.53 ± 0.60 for CACS > 0
Forget et al. 2017 [14]	Belgium	For health care,<br>Median 38 years (21-66 years)	413	1.65 ± 1.96
Wu et al. 2019 [43]	China	healthy adults<br>20-69 years	5000	1.59 ± 0.59
Fest et al. 2019 [3]	Netherlands	Rotterdam study<br>mean 56.9 years	14,926	1.30 > (n=1799)<br>1.30-1.59 (n=1747)<br>1.30-1.91 (n=1685)<br>1.92-2.41 (n=1745)<br>2.41 < (n=1739)
Rodríguez-Rodríguez et al. 2020 [11]	Spain	Noninstitutionalized older adults<br>66.2 years	1, 747	2.03 ± 0.89<br>male 2.15 ± 0.96<br>women 1.93 ± 0.82
Accardi et al. 2024 [39]	Sicilian	Healthy<br>Adult 65 ><br>Older 90 >	Adults 91<br>Older 76	1.65 ± 0.64<br>1.98 ± 0.84
Calixte et al. 2024 [38]	United States	NAHANES (2009-2016)	16,849	2.16 for all<br>2.27 (2.22-2.31) for Non-Hispanic white<br>1.78 (1.74-1.83) for Non-Hispanic Black<br>2.10 (2.04-2.16) for Hispanic<br>2.03 (1.98-2.08) for Other
Sun et al. 2025 [44]	China	Chinese residents<br>56.7 years	21,775	1.7 ± 0.8

NLR; neutrophil-to-lymphocyte ratio, NHANES; National Health and Nutrition Examination Surve, CACS; coronary artery calcium score.
